# Drug-Related Problems and Associated Factors among Patients Admitted with Chronic Kidney Disease at Jimma University Medical Center, Jimma Zone, Jimma, Southwest Ethiopia: A Hospital-Based Prospective Observational Study

**DOI:** 10.1155/2019/1504371

**Published:** 2019-10-20

**Authors:** Aster Wakjira Garedow, Eshetu Mulisa Bobasa, Amare Desalegn Wolide, Fantu Kerga Dibaba, Fanta Gashe Fufa, Birtukan Idilu Tufa, Serkadis Debalke, Kabaye Kumela Goro

**Affiliations:** ^1^Jimma University, School of Pharmacy, Jimma, Ethiopia; ^2^Jimma University, School of Biomedical Sciences, Jimma, Ethiopia; ^3^Jimma University, School of Midwifery and Nursing, Jimma, Ethiopia; ^4^Jimma University, School of Medical Laboratory, Jimma, Ethiopia

## Abstract

**Background:**

There is an alarming rise of chronic kidney disease prevalence globally associated with significant morbidity and mortality necessitating special attention as one of the major growing public health problems. Medication-related problems are common in hospitalized patients including chronic kidney disease and may lead to increase hospital stay and health care cost and augment the risk of morbidity and mortality.

**Objective:**

To determine prevalence of medication-related problems and associated factors among chronic kidney disease patients admitted to Jimma University Medical Center from April to September 2018.

**Methods:**

A hospital-based prospective observational study was conducted among 103 chronic kidney disease patients admitted to Jimma University Medical Center from April to September 2018. Data regarding patient characteristics, medications, diagnosis, length of hospitalization, and laboratory results were collected through review of patients' medical charts. Data were analyzed by using Statistical Package for the Social Sciences (SPSS) version 21.0. Univariate and multivariate logistic regression was utilized to assess the associations between dependent and independent variables. Statistical significance was considered at *p* value <0.05.

**Results:**

Out of 103 chronic kidney disease patients, 81 (78.6%) of patients had MDRs, on average 1.94 ± 0.873 per patient. The rate of overall MRPs was 30.95 per 100 medication orders. The most common MRPs among CKD patients were need additional drug therapy (62 (31%)), nonadherence (40 (20%)), and dose too low (36 (18%)). The most common cause of need additional drug therapy (52 (26%)) was untreated medical conditions; nonadherence (19 (9.5%)) was mostly due to that the patient/caregiver forgets to take/give the medication, and dose too low (29 (14.5%)) was mostly due to that the dose is too low to produce the desired response. Polypharmacy (AOR = 4.695, 95% CI: 1.370–16.091), number of comorbidities (AOR = 3.616, 95% CI: 1.015–1.8741), and stage of CKD (AOR = 3.941, 95% CI: 1.221–12.715) were independent predictors for MRPs.

**Conclusions:**

We have demonstrated that medication-related problems are high among chronic kidney disease patients. Marital statuses, stage of CKD, polypharmacy, and comorbidity were independent predictors for MRPs. Interdisciplinary health professionals should work to decrease the high prevalence of MRPs among chronic kidney disease patients.

## 1. Back Ground

Chronic kidney disease is one of the global health problems requiring early detection and treatment to prevent its progression [1] and associated with increased morbidity, mortality, and health care costs for both individual patients and health care system [2]. The global prevalence of CKD is estimated to be 11%–13% [3]. In sub-Saharan Africa (SSA), CKD is estimated to be 3-4 folds more than in developed countries [4] and comorbidity, implying concomitant use of many drugs, makes the management of these patients particularly challenging [5]. Medication-related problems (MRPs) are the major challenge to health care providers and they may affect morbidity, mortality, and patients' quality of life. CKD patients are on high risk for MRPs because of the polypharmacy and the impaired renal excretion [6, 7]. MRPs may lead to reduced quality of life, increased hospital stay, increased overall health care cost, and even increases the risk of morbidity and mortality. All patients' problems involving medications can be grouped into one of the seven types of MRPs. These include unnecessary drug therapy; need additional drug therapy, ineffective drug, dosage too low, adverse drug reactions (ADRs), dosage too high, and noncompliance [8, 9].

Since MRPs are very common in patients with CKD, identification, prevention, and management of these problems require a comprehensive, interdisciplinary approach [10–14]. It is estimated that the annual cost of drug-related morbidity and mortality is nearly 177 billion dollars in the United States [15]. Many drugs are eliminated by the kidneys and therefore may require dose adjustment in patients with renal impairment and dosing of all drugs, including antibiotics should be optimized and monitored so as to prevent ADR, avoid further renal injury, and to facilitate treatment outcomes [16–18]. The treatment of CKD stage V needs a large number and variety of drugs, which are linked to a number of MRPs, high cost, and short-term mortality [19–23]. A high number of prescribed medications due to a high number of comorbidities and complications associated with the disease, poor medication adherence, and frequent dosage changes may contribute to drug-related morbidity and MRPs [4, 24–29].

Identifying MRPs is a major task which could be taken care of by a clinical pharmacist in coordination with other health care providers through medication reconciliation [30–33]. On the other hand, educational intervention at discharge and follow-up of patients by the clinical pharmacists may also prevent adverse events and can improve patients' awareness of their drug therapy which in turn would improve their adherence to drug therapy [34–36]. The prevalence of CKD cases found to be significant in Ethiopia. In a developing country like Ethiopia, the role of a clinical pharmacist is much needed as there is a need to seal the existing gap in health care settings of the country [37]. The aim of this study was to determine prevalence of MRPs and associated factors among CKD patients admitted to Jimma University Medical Center from April to September 2018.

## 2. Methods and Participants

### 2.1. Study Setting and Population

The study was conducted at Jimma University Medical Center (JUMC), which is the only referral hospital for the south region, serving 20 million catchment areas. The study was conducted from April 1 to September 30, 2018, among CKD patients admitted to JUMC. Patients were eligible for inclusion if they were greater than 18 years of age and willing to give their informed consent. The dependent variable was MRPs and independent variables included patients' related factors: age, sex, BMI, educational status, residence, monthly income, marital status, and occupation; disease-related factors: stage of CKD, length of hospital stay, comorbid condition, past medical history, and reason of admission; drug-related factors: number of drugs per prescription, past medication history, and drug class.

### 2.2. Study Design

A hospital-based prospective observational study was used to determine the magnitude of medication-related problems and associated factors among CKD patients. The sample size was calculated by using simple proportion formula with estimated prevalence of MRPs among CKD patients, *p*=81.5% [21], 95 confidence interval, and sample error of 5%, *n* = 231. 180 CKD patients admitted to JUMC in 2017. The final sample size, 103, was calculated using the correction formula.

### 2.3. Data Collection Procedures

Well-designed questionnaires were prepared after reviewing different literatures. The questionnaire was translated from English to local language Afan Oromo and Amharic and back to English by a licensed linguist. Semi-structured interview was conducted to record patients' sociodemographic data, past medical and medication history, date of admission/discharge, and allergy/ADR history and the patient chart to collect comorbidities, current and discharge medications, and laboratory investigations. All drugs which were prescribed for CKD patients were recorded and evaluated for presence, types, and patterns of MRPs. Identified MRPs were recorded and classified using MRP registration format which was taken from *Pharmaceutical Care Practice: The Clinicians Guide* [8]. MRPs were categorized by type and medication class. Pretest was done to ensure the validity of the tools.

### 2.4. Data Processing and Statistical Analysis

Data were checked for completeness, grouped, then entered to EpiData version 4.2.0.0 software, and exported to the Statistical Package of the Social Sciences (SPSS) version 21.0 for analysis. Descriptive statistics like mean and percentage were used to present sociodemography and clinical characteristics of participants. Binary logistic regression was used to see the association between independent and dependent variables, and variables with a *p* value <0.25 were a candidate for multivariate analysis and those variables with a *p* value <0.05 were considered as significant in multivariate analysis. The ADR was assessed by using the Naranjo ADR Probability Scale [38]. Subsequently, the appropriateness of drug therapy was evaluated using the 2014 Ethiopian Standard Treatment Guideline, UpToDate, Clinical Practice Recommendations for Primary Care Physicians and Health care Providers, the KDIGO 2012 Clinical Practice Guideline, and the WHO guideline. Identified MRPs were recorded and classified using MRP registration format which was taken from Pharmaceutical Care Practice [39]. Then, the possible intervention measures were proposed and communicated with either the internist/resident/senior physician or the patient in order to resolve or prevent MRPs.

### 2.5. Ethical Consideration

Institutional review board approval was obtained from Jimma University and written informed consent was obtained from each study participant.

### 2.6. Operational Definitions

#### 2.6.1. Chronic Kidney Disease

CKD is defined as abnormalities of the kidney structure or function, present for 3 months, with implications for health [30].

#### 2.6.2. Stages of CKD

  Stage I: kidney damage with normal or increased GFR (≥90% mL/min/1.73 m^2^)
  Stage II: kidney damage with a mild decrease in GFR (60–89% mL/min/1.73 m^2^)
  Stage III: moderate decrease in GFR (30–59 mL/min/1.73 m^2^)
  Stage IV: severe decrease in GFR (15–29 mL/min/1.73 m^2^)
  Stage V: kidney failure (<15 mL/min/1.73 m^2^) [30]

#### 2.6.3. Comorbidity

Diseases or disorders that exist together with an index disease, or co-occurrence of two or more diseases or disorders in an individual.

#### 2.6.4. Social Drug Use

Use of alcohol, cigarette smoking, and chewing khat for one or more than one year.

#### 2.6.5. Polypharmacy

Use of five or more medication concomitantly.

#### 2.6.6. MRPs

Events involving drug treatment that are actually or potentially harmful to a patient's health or prevent patients to optimally benefit from treatment.

#### 2.6.7. Need Additional Drug Therapy

Additional drug therapy is required to treat or prevent a medical condition or illness from developing.

## 3. Result

### 3.1. Sociodemographic Characteristics of the Study Participants

During the six month study period, 103 CKD patients were included. Most of the study participants were in age group of 18–40 years with a mean age of 45.83 ± 17.7. Majority of the study participants 72 (69.9%) were males, 65 (63.5%) had no regular income, 66 (64.1%) were living in rural area, and 73 (70.9%) were married. Most of the study participants were 53 (51.5%) farmers and 34 (33.0%) had secondary education (Table 1).

### 3.2. Clinical Characteristics

Majority of the study participants (69 (66.99%)) had <5 comorbidities, 90 (87.4%) were newly diagnosed CKD patients, 66 (64.1%) received <5 drugs per prescription, and 80 (77.7%) were stay in hospital for ≥7 days. Most of the study participants 44 (42.7%) had normal BMI. The prevalence of CKD in the study population according to the KDIGO classification was as follows: 2 (1.9%) were in CKD stage II, 18 (17.5%) were in CKD stage III, 16 (15.5%) were in CKD stage IV, and 67 (65%) were in CKD stage V (Table 2).

**Figure 1:**
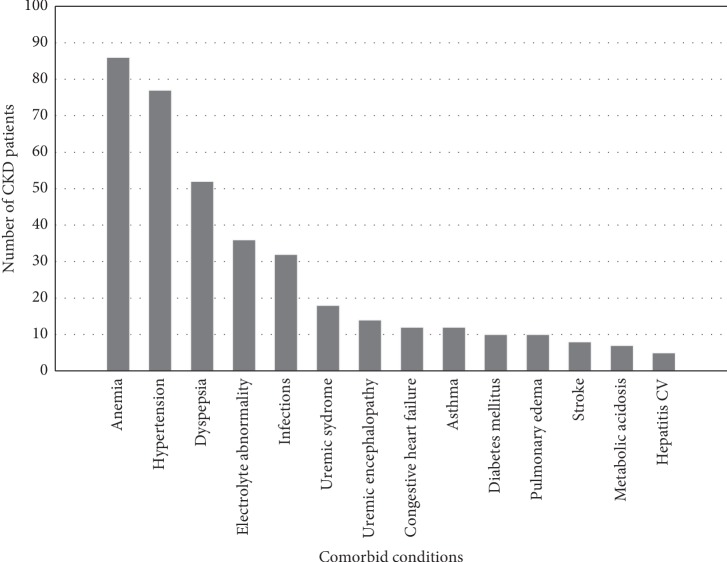
Common comorbidities among study participants at JUMC, Jimma Zone, Jimma, Southwest Ethiopia, from April to September 2018.

All of the patients were found to have one to seven comorbidities. The top five comorbid conditions were anemia (86 (83.5%)), hypertension (77 (74.8%)), dyspepsia (52 (50.5%)), electrolyte abnormality (36 (34.95%)), and infections (32 (31.1%)) (Figure 1).

### 3.3. Prevalence of Medication-Related Problems

Out of 103 study participants, 81 (78.6%) of patients had MRPs, on average 1.94 ± 0.873 MRPs per patient. The rate of overall MRPs was 30.95 per 100 medication orders. The most common MRPs among CKD patients were need additional drug therapy (62 (31%)) and nonadherence (40 (20%)). The most common cause of need additional drug therapy was untreated medical conditions (52 (26%)), while for nonadherence, the patient/caregiver forgets to take/give the medication (19 (9.5%)) was the most common cause (Table 3).

A total number of 646 medications were prescribed, and the mean number of prescribed medications per patient was 6.26 ± 1.85. From these, 219 drugs were involved in 200 different types of MRPs. The most common drug classes associated with the occurrence of MRPs among study participants include cardiovascular medications (31.9%), gastrointestinal (19.1%), and analgesic (19.1%) (Table 4).

### 3.4. Intervention for Medication-Related Problems

A total of 218 clinical interventions were undertaken at three levels of the intervention: prescriber level: 88 (40.4%); patient/career level: 56 (25.7%), and drug level: 74 (33.9%). Out of these interventions, 178 (81.6%) were accepted and 174 (79.8%) were totally solved (Table 5).

### 3.5. Predictors of Medication-Related Problems

The association of independent variables with dependent variables was investigated using both univariate and multivariate logistic regression techniques. In univariate logistic regression analysis age, marital status, length of hospital stay, social drug use, number of comorbidities, place of residence, polypharmacy, monthly income, and stage of CKD were associated with MRPs. Those variables with a *p* value <0.25 in bivariate analysis were introduced to multiple logistic regression. The result of the multivariate analysis showed participants who were married were 62% times more likely to have MRPs compared to those who were single (=AOR = 0.383, 95% CI: 0.042–0.792, *p*=0.023). Participants who took polypharmacy were 4.695 times more likely to have MRPs compared to those who did not took polypharmacy (AOR = 4.695, 95% CI: 1.370–16.091). Participants who have ≥5 comorbidities were 3.616 times more likely to have MRPs compared to those who have <5 comorbidities (AOR = 3.616, 95% CI: 1.015–1.8741). Participants who were treated for stage V CKD were 3.941 times more likely to have DRPs compared to those in other stages of CKD (AOR = 3.941, 95% CI: 1.221–12.715) (Table 6).

## 4. Discussion

In our study, most of the study participants 90 (87.4%) were newly diagnosed CKD patients (within 3 months of study enrollment) and 67 (65%) were in CKD stage V. The reasons for this were most study participants live in rural areas, had a history of many medical conditions, did not take their medications properly, and came to JUMC after developing ESRD. We found a high frequency of MRPs in 81 (78.6%) patients among CKD patients with an average of 1.94 ± 0.873 MRPs per patient. The rate of overall MRPs was 30.95 per 100 medication orders. This result is lower than the result obtained from studies conducted in Indonesian (42.7 MRP per 100 medication orders) [2] and higher than the result found in USA (6.58 MRPs per 100 medication orders) [14]. Each study participant had at least one type of MRPs and the number of MRPs per participant ranges between 2 and 4. Conversely, a study conducted in France [40] reported that MRPs experienced by 93% to 99% of studied patients and ranged between 2 and 6 MRPs per patient. These discrepancies might be due to differences in the study population, study period, and study setting.

Our analysis on MRPs showed need additional drug therapy (31% of all MRPs), which is almost similar to the studies conducted in France (30%) [13, 27]. In contrast to this finding, need additional drug therapy accounted for a larger proportion of the MRPs in studies conducted in USA (61.5%) [14], India, (40.6%) [41], Canada (51.3%) [42], and Pakistan (40.19) [6]. This is because of more comorbidities and complex CKD management algorithm identified in most study settings. However, the finding of this study was greater than the results obtained from midwestern America (17.5%) [14], USA (16.9%) [4], France (24.1%) [43], and Switzerland (18%) [44]. Dose too low accounted for 18% of all MRPs identified, which was consistent with studies conducted in France (19%) [27] and Nigeria (20.9%) [7]. This may be due to the similar study design used. Conversely, dose too low accounted for higher proportion of the MRPs in studies conducted in USA (33.5%) [19], France (25.5%) [13], Pakistan (31.1%) [6], and Beirut (10%) [45]. The difference can be explained by the difference in study design used and health care setting in which the studies were conducted.

Overdosage was accounted for 14.5% of all MRPs identified, which was consistent with studies conducted in Canada (13.6%) [42] and Switzerland (16%) [44]. Conversely, dose too high accounted for a larger proportion of the DRPs in studies conducted in USA (20.3%) [4], France (42.2%) [13], Canada (23.7%) [46], and Beirut (28%) [45]. This difference might be an underestimate due to the lack of comprehensive documentation at the point of admission in this study. However, this study's finding was higher than what was found in Netherlands (5%) [20] and Singapore (3.1%) [47]. Inappropriate drug monitoring and failure of adjusting renal-dosed drugs as per the renal function could be the reason. Another common aspect of MRPs is unnecessary drug therapy 4.8%, which was consistent with studies conducted in Canada (5.5%) [42]. In comparison, this finding is lower than the studies conducted in America (30.9%), [14], Netherlands (25.5%), [20], Switzerland (10%) [48], Nigeria (36.75%) [7], Malaysia (20.9%) [49, 50], and Pakistan (21.2%) [6]. Despite the difference of figures in many countries, polypharmacy is common among CKD population due to the nature of its management.

Ineffective drug therapy contributed for 10% of all MRPs identified. In comparison, studies conducted in India, (40.6%) [41], Canada (51.3%) [42], Beirut (28%) [45], and USA (14.9%) [4] showed larger proportion of ineffective drug therapy. This might be due to in developed countries CKD patients treated for many years which can result in drug resistance, while most of the study participants in this study were newly diagnosed patients. ADR (2%) is another important subset of MRPs identified. Conversely, studies conducted in USA (20.7%) [19], India (40%) [41] Canada (6.8%) [42], and Singapore (25%) [47] had higher ADR of all MRPs. The difference in this prevalence could be explained by the fact that the other studies were carried out over a long period thus identify more ADRs. Nonadherence was accounting for 20% of all MRPs identified, which was higher than the studies conducted in USA (16.9%) [51], Netherlands (5.6%), [20], and India (11.1%) [39]; however, lower than what was found in Canada (28%) [42] and Singapore (28.1%) [47]. These findings can be attributed to failure of the patients to understand their disease process and the benefits of adhering to medications as prescribed. Indeed, a study in France established an obvious lack of knowledge concerning CKD and its treatment objectives which led to a potential for nonadherence [21]. The high number of drugs per participant as well as comorbidities could also contribute to the high prevalence of nonadherence [52].

Marital status was significantly associated with the number of MRPs which is different from most findings [4, 27, 42] where marital status was not an independent predictor of MRPs. This difference might be due to marital status difference of study participants and large sample of the current study. Polypharmacy was significantly associated with a number of MRPs which is similar with studies conducted in France and India [27, 42, 53]. The number of comorbidities was significantly associated with the number of MRPs which is in agreement with studies conducted in USA and India [14, 41]. Possible reasons for prescribers not picking up some of the comorbidities and inadequate information transfer between the patient and the prescriber. Stage V CKD patients were significantly associated with the number of MRPs which is consistent with the study conducted in Norway [43]. This was due to CKD stage V patients are known to suffer from numerous comorbidities and complications. As a result, the treatment needs a large number and variety of drugs [21].

A total of 218 clinical interventions were undertaken at three levels of the intervention. Out of these interventions, 178 (81.6%) were accepted, 174 (79.8%) were totally solved, and 40 (18.3%) MRPs were partially solved due to lack of cooperation of prescriber and patient. Amongst the MRPs indentified, 78.1% were successfully resolved. Conversely, a study conducted in Nigeria [7] showed the acceptance of clinical interventions was 67.54%, and 7.86% was successfully resolved.

## 5. Conclusion

We have demonstrated that MRPs are high in CKD patients. Marital statuses, stage of CKD, polypharmacy, and comorbidity were independent predictors for MRPs.

### 5.1. Recommendations

JUMC should implement strategies to decrease the high prevalence of MRPs among CKD patients and should assign an interdisciplinary health care provider in inpatient settings to decrease the prevalence of MRPs among CKD patients. Since CKD patients are at high risk to present with comorbidities, physicians should consider all while treating them. The health care provider should adhere to dose adjustment recommendationsand should prescribe essential medicines only to reduce polypharmacy, and if possible, polypharmacy should be avoided. CKD patients should adhere to their medications.

## Figures and Tables

**Table 1:** Sociodemographic characteristics of CKD patients at JUMC, Jimma Zone, Jimma, Southwest Ethiopia, from April 01 to September 30, 2018.

Variable		Frequency
Age (in yrs)	18–40	46 (44.7)
	41–60	31 (30.1)
	>60	26 (25.3)

Sex	Male	72 (69.9)
	Female	31 (30.1)

Marital status	Single	
	Married	

Occupation	Farmer	53 (51.5)
	Unemployed	31 (30.1)
	Employed	12 (11.7)
	Merchant	7 (6.8)

Monthly income	No regular income	65 (63.1)
	≥1000	38 (36.9)

Place of residence	Rural	66 (64.1)
	Urban	37 (35.9)
	No formal education	32 (31.1)

Educational status	Primary school	25 (24.3)
	Secondary school	34 (33.0)
	College/university	12 (11.7)

Social drug use	Nonuser	53 (51.4)
	User	50 (48.5)

**Table 2:** Clinical characteristics of CKD patients at JUMC, Jimma Zone, Jimma, Southwest Ethiopia, from April 01 to September 30, 2018.

Variables		Frequency
Number of comorbidities	<5	69 (66.9%)
	≥5	34 (32.9%)

BMI	≤18	38 (36.9)
	18.5–24.5	44 (42.7)
	25–29.9	17 (16.5)
	≥30	4 (3.9)

Number of drug taken per day	<5	55 (53.3)
	≥5	48 (46.6)

Duration with CKD (yrs)	<1	90 (87.4)
	≥1	13 (12.6)

Length of hospital stay	<7 days	23 (22.3)
	≥7 days	80 (77.7)

Stage of CKD patients	2	2 (1.9)
	3	18 (17.5)
	4	16 (15.5)
	5	67 (55)

**Table 3:** Prevalence of MRPs and their causes among CKD patients at JUMC, Jimma Zone, Jimma, Southwest Ethiopia, from April 1 to September 30, 2018.

MRPs domain	DRP subdomain	Cause	Frequency	
Indication	Need additional drug therapy	A medical condition requires the initiation of drug therapy	52 (26)	62 (31)
		Preventive drug therapy is required to reduce the risk of developing a new condition	10 (5)	
	Unnecessary drug therapy	There is no valid medical indication for the drug therapy at this time	5 (2.5)	9 (4.5)
		Multiple drug products are being used for condition that requires single-drug therapy	4 (2)	

Effectiveness	Ineffective	The drug is not the most effective for the medical problem	12 (6)	20 (10)
		The medical condition is refractory to the drug product	8 (4)	
	Dose too low	The dose is too low to produce the desired response	32 (16)	36 (18)
		The dosage interval is too infrequent to produce the desired response	4 (2)	

Safety	Adverse drug reaction	The drug product causes an undesirable reaction that is not dose-related	3 (1.5)	4 (2)
		A drug interaction causes an undesirable reaction that is not dose-related	1 (0.5)	
	Dose too high	Dose is too high	19 (9.5)	29 (14.5)
		The dosing frequency is too short	10 (5)	

Compliance	Noncompliance	The patient/caregiver does not understand the instructions	5 (2.5)	40 (20)
		The patient/caregiver prefers not to take/give the medication	12 (6)	
		The patient/caregiver forgets to take/give the medication	19 (9.5)	
		The drug product is too expensive for the patient	4 (2)	

**Table 4:** Classes of medications commonly prescribed and related with MRPs among study participants at JUMC, Jimma Zone, Jimma, Southwest Ethiopia, from April 1 to September 30, 2018.

Therapeutic classes	Frequency (%), *N* = 646	Frequency of MRPs (%), *N* = 219
Cardiovascular drugs	172 (26.6)	70 (31.9)
Anti-infective	89 (13.8)	42 (19.1)
Gastrointestinal drugs	124 (19.2)	42 (19.1)
Analgesic	82 (12.7)	40 (18.3)
Drugs for blood disorder	40 (6.2)	14 (6.3)
Fluid and electrolytes	82 (12.7)	6 (2.7)
Endocrine drugs	38 (5.9)	3 (1.4)
Respiratory drugs	19 (2.9)	2 (0.9)

**Table 5:** Interventions and outcomes of interventions of MRPs among study participants at JUMC, Jimma Zone, Jimma, Southwest Ethiopia, from April 1 to September 30, 2018.

	Interventions	Frequency (%), *N* = 218
At prescriber level	Prescriber informed only	2 (0.9)
	Prescriber asked for information	1 (0.5)
	Intervention proposed to prescriber	50 (22.9)
	Intervention discussed with prescriber	35 (16.1)

At patient level	Patient (drug) counseling	54 (24.7)
	Patient referred to prescriber	1 (0.5)
	Spoken to family member/care giver	1 (0.5)
At drug level	Drug changed	9 (4.1)
	Dosage changed	24 (11)
	New drug started	38 (17.4)
	Drug stopped	3 (1.4)

Intervention acceptance	Intervention accepted	178 (81.6)
	Intervention not accepted	40 (18.3)

Outcome of interventions	Problem totally solved	174 (79.8)
	Problem partially solved	40 (18.3)
	Problem not solved	8 (3.6)

**Table 6:** Binary logistic regression result of predictors of MRPs among CKD patients at JUMC, Jimma Zone, Jimma, Southwest Ethiopia, from April 1 to September 30, 2018.

Predictor variable	Category	DRPs		COR (95% CI)	AOR (95% CI)	*p*-value
		Yes	No			
Age	<50	43	12	1.00	1.00	0.270
	≥50	38	10	0.926 (0.357–2.400)	0.855 (0.241–3.029)	

Sex	Male	57	16	1.146 (0.441–2.977)		0.816
	Female	24	7	1.00		

BMI	≤18	28	10	1.00		
	18.5–24.5	34	10	0.129 (0.345–5.875)		0.772
	25–29.9	15	2	0.106 (0.129–12.129)		0.918
	≥30	3	1	0.080 (0.106–12.557)		0.999

Duration since diagnosis with CKD in (yrs)	<1	82	8	1.00		0.634
	≥1	15	6	1.816 (0.156–21.117)		

Length of hospital stay	<7 days	32	9	1.000	1.000	0.077
	≥7 days	49	13	2.320 (1.345–3.543)	2.720 (2.325–3.543)	

Marital status	Married	53	9	1.00	1.00	0.023
	Single	28	13	0.360 (0.136–0.953)	0.383 (0.042–0.792)	

Educational status	No formal education	22	7	1.00	1.00	0.068
	Primary school	23	5	0.738 (0.246–2.218)	0.234 (0.049–1.115)	0.291
	Secondary school and above	36	10	1.294 (0.383–4.371)	0.415 (0.081–2.123)	0.251

Monthly income	No regular income	51	14	1.00		0.166
	≥1000	30	8	0.800 (0.291–2.199)		

Place of residence	Urban	28	9	1.00	1.00	0.252
	Rural	53	13	1.00	2.460 (0.713–8.495)	

Stage of CKD	II, III and IV	29	7	0.505 (0.112–1.457)	1.00	0.022
	V	52	15	1.114 (0.404–3.074)	3.941 (1.221–12.715)	

Social drug use	User	39	11	1.00	1.00	0.272
	Nonuser	42	11	0.750 (0.289–1.944)	0.510 (0.153–1.696)	

Number of medications	<5	48	7	1.00	1.00	0.013
	≥5	33	15	3.871 (1.366–10.994)	4.695 (1.370–16.091)	

Number of comorbidities	<5	53	16	1.00	1.00	0.043
	≥5	28	6	2.029 (0.77–5.350)	3.616 (1.015–1.874)	

## Data Availability

The data used to support the findings of this study are available from the corresponding author upon request.

## Abbreviations

ADR: Adverse drug reaction
AKI: Acute kidney injury
CCB: Calcium-channel blocker
CKD: Chronic kidney disease
CVD: Cardiovascular disease
DRPs: Drug-related problems
ESRD: End-stage renal disease
GFR: Glomerular filtration rate
IWD: Indication without drug
JUMC: Jimma University Medical Center
KDIGO: Kidney Disease Improving Global Outcomes
RI: Renal impairment
SPSS: Statistical Package for the Social Sciences
SSA: Sub-Saharan Africa
USA: United States of America.
